# How to Survive at Point Nemo? Fischer–Tropsch, Artificial Photosynthesis, and Plasma Catalysis for Sustainable Energy at Isolated Habitats

**DOI:** 10.1002/gch2.202300086

**Published:** 2023-12-04

**Authors:** lgor Levchenko, Shuyan Xu, Oleg Baranov, Kateryna Bazaka

**Affiliations:** ^1^ School of Engineering, College of Engineering, Computing and Cybernetics The Australian National University Canberra ACT 2600 Australia; ^2^ Plasma Sources and Application Centre, NIE Nanyang Technological University Singapore 637616 Singapore; ^3^ Department of Theoretical Mechanics Engineering and Robomechanical Systems National Aerospace University Kharkiv 61070 Ukraine; ^4^ Department of Gaseous Electronics Jozef Stefan Institute Ljubljana 1000 Slovenia

**Keywords:** artificial photosynthesis, Fischer–Tropsch, plasma catalysis

## Abstract

Inhospitable, inaccessible, and extremely remote alike the famed pole of inaccessibility, aka Point Nemo, the isolated locations in deserts, at sea, or in outer space are difficult for humans to settle, let alone to thrive in. Yet, they present a unique set of opportunities for science, economy, and geopolitics that are difficult to ignore. One of the critical challenges for settlers is the stable supply of energy both to sustain a reasonable quality of life, as well as to take advantage of the local opportunities presented by the remote environment, e.g., abundance of a particular resource. The possible solutions to this challenge are heavily constrained by the difficulty and prohibitive cost of transportation to and from such a habitat (e.g., a lunar or Martian base). In this essay, the advantages and possible challenges of integrating Fischer–Tropsch, artificial photosynthesis, and plasma catalysis into a robust, scalable, and efficient self‐contained system for energy harvesting, storage, and utilization are explored.

## Graphical abstract

Extremely remote places like pole of inaccessibility called Point Nemo, remote islands or outposts on Mars and Moon are difficult to settle. The critical challenge is the stable energy supply. In this essay, the possible architecture and challenges of integrating Fischer–Tropsch, artificial photosynthesis, and plasma catalysis into an efficient self‐contained system for energy harvesting, storage, and utilization are explored.

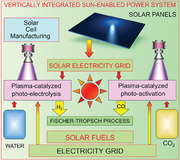

## 1 Introduction

**Figure 1:**
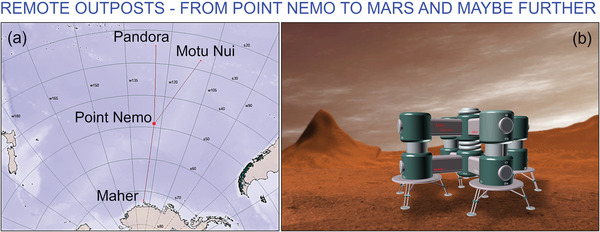
Examples of “Points Nemo” on Earth and beyond? The actual Point Nemo, also known as “the oceanic pole of inaccessibility,” can be found at 48°52.6′S 123°23.6′W, and is regarded as the location most physically removed from any body of land (a). Indeed, one would need to cover at least a thousand miles of ocean in order to reach land, regardless of direction of travel. Other examples of similarly isolated poles and point of inaccessibility include the Eurasian pole of inaccessibility (EPIA) in the Gurbantünggüt Desert of China, which also happens to be the furthest point on land from the ocean; as well as the poles of accessibility for individual continents. While used metaphorically here to highlight remoteness and isolation, even less remote locations are difficult to settle. The logistics of providing sustained access to energy and input resources is one of the critical factors in determining the success of such settlements. The issue of transport becomes one of feasibility when extra‐terrestrial locations, e.g., on Moon and Mars are considered due, especially for heavy traditional systems. Reproduced under the terms of the CC BY‐SA 4.0 license.^[^ ^4^ ^]^ (b) Artist's impression of a Mars settlement, reprintred under terms of the CC BY license.^[^ ^5^ ^]^

Energy is the alpha and omega for colonizing physically isolated environments, the *Point Nemos* ^[^ ^1^, ^2^ ^]^ of this Earth and beyond. In our modern world, we have grown to rely heavily on fossil fuels to give us light and heat, and to power our machines. And even those devices we have built to harvest and use the energy of the nature are largely mined and manufactured using the energy from fossil fuels. Yet, if we consider small, remote habitats on an isolated island, deep in an Antarctic or Sahara desert or in a jungle, we have to think beyond the fossil fuel‐enabled paradigm. This is because the cost of building physical infrastructure to either deliver or to locally extract and process fossil fuels would be prohibitive, if at all feasible, and for some habitats, e.g., those on the Moon or Mars, these would not be possible (**Figure** **1**).^[^ ^1^, ^3^ ^]^

When considering fossil fuel alternatives, solar energy seems like a natural choice, given its relative abundance, omnipresence and reliability. We are also very familiar with this source of energy, many of us choosing to use it in our houses and places of business to reduce our carbon footprint. Yet, for us it remains a choice. All the critical infrastructure that we rely on for our survival, from hospitals to transportation and communications to food storage, is supported by multiple sources of power. In contrast, under resource‐constrained conditions of an isolated habitat, having multiple layers of reliable power supply, such as the electrical power grid and fuel generators that most of us are accustomed to, is not feasible, and reliability of systems used for energy generation, storage, and conversion becomes truly critical to one's survival. Similarly, efficiency and lifetime of harvesting and storage devices also become paramount when one considers sunlight availability cycles in the polar regions of Earth, or the far less predictable intense Martian dust storms that often reach the size of a continent, at times growing into planet‐encircling dust storms, and last for weeks. It also goes without saying that such systems need to be compact, light and portable for ease of transportation, as well as robust and simple enough for non‐experts to assemble, operate, and maintain.

**Figure 2:**
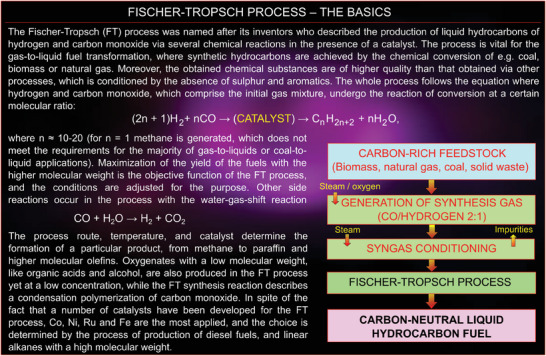
The basics of the Fischer–Tropsch technology, where carbon‐rich feedstock (gas, coal, or biomass) is first converted into a mixture of hydrogen and carbon monoxide called Syngas, and then processed in the presence of a catalyst into liquid fuels made of hydrocarbon chains.^[^ ^17^, ^18^ ^]^ The Fischer–Tropsch process is a key stage in the whole technological chain of converting carbon‐containing substances into hydrocarbon fuels, where latter can be considered as mass‐ and cost‐efficient energy storage media—much more efficient than electric batteries. The technological chain of reactions is shown in the diagram. Water and CO_2_ splitting using, e.g., an artificial photosynthesis‐based system and plasma are other important elements of the proposed energy system.

Energy utilization is the final piece of the energy puzzle that we would like to focus on in this essay. Scarcity of one or more process inputs is the likely reality of these habitats—be it water, air, energy, or natural resources. Hence, there is a necessity for processes to maximize quality and yield while minimizing consumption and waste. Processes that are more indiscriminate in the quality or nature of the input feedstocks yet capable of producing reasonable quality outputs (like the *Fischer–Tropsch technology for the production of liquid fuels from simple gases*,^[^ ^6^, ^7^ ^]^ see **Figure** **2**) would win over processes that demand high purity of inputs or very complex processing environments to deliver a comparable, be it a better product that fulfils the same function.^[^ ^8^, ^9^ ^]^ Ideally, of course, we would like to develop process that deliver on all fronts.^[^ ^10^, ^11^ ^]^ One way to do it is by harnessing the increased chemical reactivity of the highly energetic fourth state of the matter, *aka plasma*.^[^ ^12^, ^13^ ^]^ Plasma is the dominant form of baryonic matter in our Universe, and when encountered in the outer layer of the stars, plasma is extremely hot, with temperature reaching millions of degrees. However, under certain conditions, it is possible to push the gaseous matter into the plasma state without substantially increasing its bulk temperature, while benefiting from the significantly increased chemical reactivity that can tremendously speed up the processing time and even sustain reactions that would not happen otherwise.^[^ ^14^ ^]^ And certainly, the *artificial photosynthesis* is one of the promising candidates for the advanced self‐sustained energy systems.^[^ ^15^, ^16^ ^]^

*So, how do we go about integrating all of the above into a single energy ecosystem for remote habitats?*

## 2 Design Philosophy

### 2.1 Understand Constraints

Before we even embark on the discussion about the possible designs and pathways for their integration into a single system, we need to define the nature of the remote habitat we are designing for. Some of these are easier to define, e.g., we have substantial quantity of data about building and operating polar and desert stations. Indeed, over the history of humanity, the incessant, almost written into our genome drive to go far and wide has led many an explorer to venture into the lands unknown, providing us with an abundance of life experience about survival under austere conditions of barren deserts and tiny islands lost at sea.

In contrast, while the extraterrestrial missions have always attracted pioneers, we have only relatively recently reached a point in our technological development when extraterrestrial bases are becoming a reality. This means that when it comes to habitat design, our knowledge base and practical experience is far more limited when compared to that on Earth, and undoubtedly we are likely to make many mistakes when our assumptions and modelling efforts turn out to be wrong, which is of course part and parcel of doing something this radically new. Yet, many believe our desire to settle beyond the bounds of our planet will ensure both our development and our survival as a civilization.

Indeed, those behind the development of the Mars Express spacecraft have long sustained a belief that it might be possible to find undersurface and subglacial liquid water.^[^ ^19^ ^]^ Furthermore, similar to Earth, Mars is expected to have substantial mineral resources at and under its surface layer, with a recently confirmed evidence of metal ores and other vital mineral substances.^[^ ^20^ ^]^ These features of the Red Planet have firmly cemented its status as an ultimate space colonization destination for near future,^[^ ^21^ ^]^ with the upcoming ambitious ideas of future Mars terraforming.^[^ ^22^ ^]^

However, before we endeavour to send colonists to Mars, or embark on other equally ambitious missions,^[^ ^23^ ^]^ we need to prove our ability to inhabit our near‐Earth space safely and effectively. “*The Moon is the proving ground for our eventual mission to Mars*,” NASA administrator Jim Bridenstine said at a conference in May 2019.^[^ ^24^ ^]^ And as previously mentioned, this is not a trivial task, despite the relative proximity of this target and availability of many useful technologies that can be used for lunar colonization. For instance, the evident absence of gaseous atmosphere, and hence precipitation or cloud cover that often limit solar energy harvesting on Earth, renders the surface of the Moon seemingly ideal for the use of solar‐driven technologies. Yet, the very same phenomena may lead to device overexposure to both excess sunlight and heat, reducing the device lifetime and promoting device failure. Hence, it is critical to understand the spatio‐temporal distribution of solar irradiance and diurnal insolation so that solar harvesters, research (and possibly, in future, mining and manufacturing) facilities, and human habitats can be located in areas that would minimize the detrimental impact of overexposure while maximizing useful energy generation. We also need to think outside the box when it comes to thermal management of equipment and structures that are being directly exposed to cosmic radiation. Regolith, a layer of unconsolidated, heterogeneous surface deposit that covers solid lunar rock, is a low‐density material with low thermal inertia and low thermal conductivity, which is particularly true for its top‐most layer. It is so effective that beyond the depth of half‐meter to a meter, the temperature remains almost constant (arising from the satellite's internal heat flux rather than insolation), and as such, regolith has been considered as a possible locally sourced solution for large area thermal and radiation protection. This will be very useful given that heat exchange under vacuum conditions is severely limited.^[^ ^25^ ^]^ At the same time, the high temperature heat may be effectively harnessed to drive thermochemical reactions for local manufacturing or resource extraction and conversion.

Interestingly, many of the technologies that we design for space^[^ ^26^ ^]^ are making us better at colonizing remote areas on Earth. For one, we are able to use space technologies to study the remote location in an unprecedented detail before embarking on the settlement journey. As such, we can be better prepared for the environment we enter. Secondly, technologies designed for space have extremely stringent requirements when it comes to compactness, weight, efficiency, lifetime—all the key attributes of a well‐designed system for terrestrial settlement. And while one may ask why, if we stay on Earth, do we even need to explore desserts and oceans, one should be reminded that most of the world's surface is covered in water, in the form of oceans. With the total landmass of less than 30%, an estimated third of which is desert, it appears that we are utilizing a very a small fraction of the resource that is available to us, with more than 80%of the Earth surface remaining largely unfit for habitation at the standard most of us are used to. Clearly, with a growing number of global challenges the humanity is facing, from climate change to inequality of access to resources, settlement of currently underutilized areas may provide a pathway for relieving the burden on the overpopulated and overexploited areas of Earth, and possibly lessen our negative impact on local ecosystems.

### 2.2 Define Critical Features

Regardless of whether settling isolated locations on Earth or in space, energy systems will need to be implemented as the first priority, being critical to every aspect of the mission—from heating and cooling shelters, to extracting fresh water from oceans and wastewater, to producing oxygen from CO_2_ in space,^[^ ^27^ ^]^ to sustaining food production and preservation, and supporting other research, extraction, or manufacturing functions one may envisage.

On the most fundamental level, an energy system for a remote habitat should be one with an essentially *self‐sustained, closed‐loop character* in that it uses the energy it generates and does so with the required degree of efficiency, reliability and safety. The elements within the system should be multi‐functional, their function scalable, and the system itself needs to be agile and flexible to be compact and economical, as well as to mitigate risks and failures through reconfiguration. The system needs to be as simple as possible for ease of maintenance and operation, and to maximize robustness and lifetime. Importantly, the system is required to cater to all energy needs of the settlement, including recycling and local production of components required for its own operations, i.e., *self‐sustainability*. Depending on the local constraints, this may include *all of the essential components* for primary energy harvesting (solar cells, solar evaporative systems,^[^ ^28^ ^]^ etc.), inclusive of the processes for the extraction, processing, and synthesis of essential materials and chemical reagents, and their assembly into devices, as well as for energy utilization, which covers all the activities related to auxiliary industries and human habitats. Moreover, the day/night cycling efficiency should be maximized given the constrains of closed, isolated habitats.

General requirements for the efficient solar energy harvesting systems, especially those designed for autonomous operation in remote areas, dictate the lowest cost per watt of the electricity supply, and the highest energy per mass ratio for the energy storage sub‐system. There has been significant progress made in the area of solar energy generation and storage—primarily driven by the need for these technologies to compete with the present‐day wholesale of electricity from fossil fuels. Indeed, to do that, the cost per watt of silicon solar cells (which share over 90% of the current market) must be lower than $1 per watt.^[^ ^29^ ^]^ The progress has been primarily sustained by the development of novel materials and nanostructured metamaterials, and new device architectures for solar cell elements.^[^ ^30^ ^]^ Currently, the solar cell industry is moving toward the development of a low‐cost, high‐performance SiN_x_ antireflective coating of graded index, and AlO_x_ rear surface passivation for higher cell efficiency. However apart from the solar cell that transform the light energy directly to electricity, different approaches are also under consideration and may adequately compete with solar cells in the nearest future.^[^ ^31^, ^32^ ^]^

### 2.3 Integrate and Optimize

With respect to the energy storage, especially when a large amount of energy need to be stored for powering, e.g., manned bases at remote outposts on Mars or in desert areas, traditional electrical approaches based on batteries and supercapacitors possessing low energy per weight (about 1–2 MJ kg^−1^), are not sufficiently cost and mass efficient. Instead, *chemical storage techniques* featuring inherently much higher energy per weight (>20 MJ kg^−1^) might be the most promising solution. Not surprisingly, extended research efforts on fuels solar‐produced via thermochemical conversion or by using photocatalytic nano‐devices have recently been initiated in Netherlands, Germany, United States, China, and several other countries.^[^ ^33^, ^34^ ^]^

However, these chemical energy storage methods, *lacking a cost‐effective catalyst*, still suffer from low conversion efficiency (<5%). This results in a low rate in, e.g., a very promising water–gas shift reaction (WGS) where CO and H_2_ can be transformed into each other.^[^ ^35^ ^]^ In the WGS technology, a parallel supply of CO and H_2_ is proposed to cater for a high efficiency, high throughput industrial demand.

Thus among the most pressing problems facing the highly efficient, self‐sustained energy systems, we could crystallize the three major ones: i) day/night cycling at the highest possible level, including, e.g., possible long nights in Antarctic, on Mars during long dust storms^[^ ^36^ ^]^ and similar conditions; ii) full self‐sustainability under the extremely limited resources; and iii) highest energy efficiency under conditions of low solar irradiance and insolation (Mars, low Sun inclination in Antarctica, dust storms in desert Earth regions).

A promising solution may be solar energy harvesting with novel highly efficient systems, and energy storage in the form of chemical fuels produced via an integrated set of innovative processes involving artificial photosynthesis via water splitting, CO_2_‐CO conversion, and the Fisher–Tropsch process.

Integration of these processes may result in a synergistically enhanced, highly sustainable, closed‐loop energy production system.

## 3 The Processes

The first step is to implement a plasma‐enabled process for the production of fuels (i.e., H_2_ and CO), where plasma takes on the role of the potentially less efficient chemical catalyst. Plasma‐catalyzed photo‐electrolysis with high‐rate production of H_2_ could be the most promising technique.^[^ ^37^ ^]^

**Figure 3:**
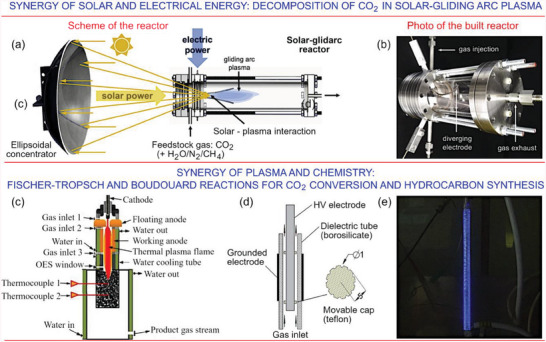
Identifying synergies for greater efficiency. Synergistic uses of (top) solar and electrical energy for CO_2_ decomposition, and (bottom) plasma and chemistry in plasma‐enabled Fischer–Tropsch and Boudouard reactions. TOP PANEL: Solar‐glidarc carbon dioxide conversion system. a) A gliding arc discharge initiated in CO_2_ is exposed to collimated solar irradiation, with additional gases injected into the system to accelerate processes conducive to CO_2_ breakdown. b) Optical photograph of the built reactor. Reproduced with permission.^[^ ^44^ ^]^ Copyright 2020, Elsevier. BOTTOM PANEL: Fischer–Tropsch and Boudouard reactions for CO_2_ conversion and hydrocarbon synthesis. Boudouard reaction is capable of producing the solid carbonous deposits out of carbon‐containing gas. c) Photo of an experimental facility used to conduct Boudouard reaction guided by a thermal plasma, where the gas mixture is introduced near the anode of the plasma‐generating circuit in a drift section. Reproduced with permission.^[^ ^46^ ^]^ Copyright 2019, Elsevier. d,e) Schematic diagram and optical photograph of the dielectric barrier discharge reactor. Reproduced with permission.^[^ ^48^ ^]^ Copyright 2019, Elsevier.

**Figure 4:**
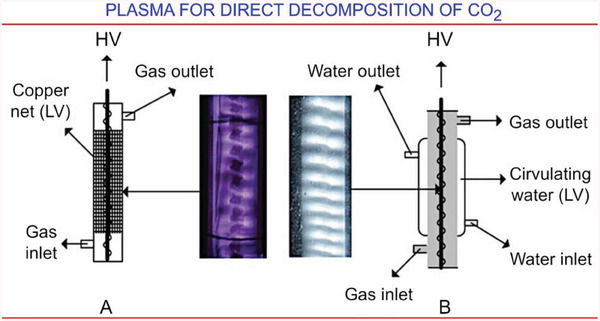
The availability of water is among key challenges in remote habitats, since virtually any sub‐system requires water for its reliable, efficient operation. This example illustrates the impact of water cooling on efficiency of CO_2_ splitting. Carbon dioxide was split in a wire‐cylinder DBD plasma setup with autogenous cooling at standard ambient temperature and pressure. The production rate reached 26.1% without changing the discharge conditions with respect to the catalyst, atmosphere, or setup configuration. Additional removal of heat via, e.g., externally reticulated water further increased efficiency and stabilized and homogenized the plasma discharge. Reactor with a conventional wire‐cylinder DBD plasma contained in Pyrex glass is denoted as “A,” while the self‐cooling DBD facility is denoted as ‘B.’ Reprinted with permission.^[^ ^45^ ^]^ Copyright 2017, Wiley.

Splitting of carbon dioxide CO_2_ → CO + 0.5O_2_ requires a rather large amount of energy to conduct this endothermic process, and that is why plasma is considered as very beneficial tool to conduct the reaction.^[^ ^38^ ^]^ However, successful direct implementation of glow,^[^ ^39^ ^]^ pulsed,^[^ ^40^ ^]^ microwave,^[^ ^41^ ^]^ arc,^[^ ^42^ ^]^ and DBD^[^ ^43^ ^]^ plasma discharges for the purpose is reported. Further development of these methods includes use of collimated solar irradiation to be adsorbed by plasma (**Figure** **3**a,b),^[^ ^44^ ^]^ self‐cooling^[^ ^45^ ^]^ of the plasma discharge (**Figure** **4**), solid carbon species (Boudouard reaction, Figure 3c)^[^ ^46^ ^]^ to increase the overall effectiveness, and quenching^[^ ^47^ ^]^ to enhance the vibrational–translational nonequilibrium and thus to affect the reaction rates.

Water splitting can potentially become one of the preferred methods for the production of abundant quantities of green hydrogen to be used as energy source, as well as oxygen that could be used for breathing in some conditions. Similarly to carbon dioxide splitting, plasma technologies are also widely used for decomposition of water. State‐of‐the‐art water splitting process such as conventional electrolysis, artificial photosynthesis, etc. still suffers from low efficiency and low throughput. On the other hand, plasma electrolysis is able to generate hydrogen gas with a much greater yield and lower energy and environmental budget when compared to typical electrolysis, showing exciting future for high rate H_2_ production. However, current limitations of plasma‐driven processes include the process controllability and excess heat generation which vaporizes water instead of splitting (which can become a significant problem for habitats where water is scarce). This may be tackled by utilizing plasma of a specific electron temperature to excite water molecules in the solar splitting process to maximize H_2_ production while minimizing energy consumption and improving process controllability. In spite of the fact that direct plasma applications such as rotating gliding arc in water vapours,^[^ ^49^ ^]^ plasma irradiation of water,^[^ ^50^ ^]^ or nitrogen plasma treatment of electrolyte^[^ ^51^ ^]^ are reported, catalyst preparation is in‐demand. Oxygen plasma is engaged to produce two‐dimensional Ni_2_P electrocatalyst,^[^ ^52^ ^]^ PH_3_ plasma is implemented to phosphorize Fe_x_‐NiCoOH precursor,^[^ ^53^ ^]^ H_2_ plasma treatment allows generating oxygen vacancies ain TiO_2_ photoanodes to suppress the recombination of carriers,^[^ ^54^ ^]^ N_2_ plasma utilization for simultaneously generation of oxygen vacancies and N‐doping of TiO_2_ nanotube photoanodes,^[^ ^55^ ^]^ to mention but a few.

The plasma catalyzed photo‐CO_2_ activation for high conversion efficiency of CO_2_ to CO is one more promising way, and nowadays plasma‐activated conversion of CO_2_ by use of catalyst to achieve renewable chemicals and fuels is a well‐developed branch of science.^[^ ^56^, ^57^ ^]^ The multi‐step CO_2_ dissociation process which involves vibrational excitation of ground state CO_2_ (1Σ+) requires the least amount of input energy and hence it is the most effective way for CO_2_ conversion. In order to promote vibrational excitation rather than electronic excitation, the input energy needs to be kept around 1 eV per molecule. As it turns out, the high‐density plasma with controllable electron cooling and confinement possesses fundamental advantages for photo‐CO_2_ activation. For Fischer–Tropsch process, plasma can be employed as for catalyst production^[^ ^58^ ^]^ as for breaking H_2_ and CO bond.^[^ ^59^ ^]^ Glow discharge was applied to decompose metal salt and produce cobalt crystallites^[^ ^60^, ^61^ ^]^ and Co/TiO_2_ catalysts,^[^ ^62^ ^]^ arc plasma is suitable synthesis of hydrocarbons from syngas, while DBD plasma allows intensification of Fischer–Tropsch process over Co/Cu catalysts^[^ ^48^, ^63^ ^]^ (Figure 2d,e). Moreover, sawdust, plastics and lignin, pyrolysis oil, and other waste products are also known as a source of high‐quality syngas and porous carbon species, when arc or microwave plasmas are applied;^[^ ^64^, ^65^ ^]^ high content of hydrogen and carbon monoxide (about 90%) can be achieved using these processes.^[^ ^66^ ^]^ Other examples include catalyst‐free production of hydrogen in solar‐driven plasmas,^[^ ^67^ ^]^ plasma‐enabled ethanol production for the further production of hydrogen,^[^ ^68^ ^]^ plasma‐assisted conversion of lignin into fuels,^[^ ^69^ ^]^ and plasma‐enabled conversion of algae into fuels.^[^ ^70^ ^]^

**Figure 5:**
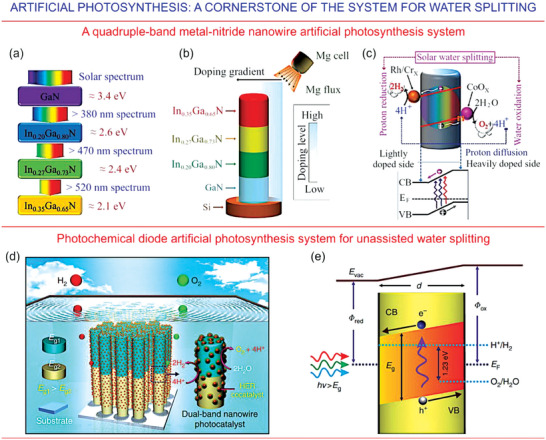
Artificial photosynthesis—one of the cornerstones of the proposed architecture for highly efficient, self‐sustained energy systems for remote habitats and extra‐terrestrial outposts. TOP PANEL: Photocatalytic water splitting by use of an assembly of nanowires made of metal nitride in neutral pH liquid media. a) A schematic to illustrate the best conditions for adsorption of solar radiation by use of the multi‐band InGaN assembly with different indium content. b) A design of the quadruple‐band InGaN nanowire, where an electric field is generated by a Mg lateral gradient doping, which is created by the Mg effusion cell tilted with respect to the nanowire; c) the field is engaged for separation of charges and their use for water redox reactions. Reproduced with permission.^[^ ^84^ ^]^ Copyright 2019, The Royal Society of Chemistry. BOTTOM PANEL: Photochemical diode artificial photosynthesis system for unassisted water splitting. d) The process of water splitting by use of double‐band nanowires with hydrogen evolution reaction catalyst dots arranged on their surfaces. Recombination or transfer of carriers is not employed in this method, as well as current matching along the non‐homogeneous vertical structure, which is in contrast to processes occurring in tandem photoelectrochemical cells or photovoltaic devices. Reactions on the side surfaces of every layer include oxidation of water and reduction of proton. e) A schematic of energy levels of the developed photochemical diode with a radial thickness *d*, where the built‐in electric field separates the electrons and holes and guides them toward the cathode and anode. Unlike the common p‐n photochemical diodes, just single photon is necessary to create an electron–hole pair to promote the redox reaction. Reproduced under the terms of the CC BY license.^[^ ^85^ ^]^

In should be stated that water splitting, CO_2_‐CO conversion, and the Fisher–Tropsch process are considered here *as the foundations of the concept of survival in isolated habitats*. Other possibilities should be implemented with respect to the necessity of creation of living conditions at some locations with limited resources of water, food, and energy. In regions with access to seawater, the use of new nanomaterial‐based technologies can provide a solution for supplying the necessary amount of drinking water, such as a setup designed for robust seawater desalination and sewage purification.^[^ ^71^ ^]^ Here, a huge surface‐to‐volume ratio accompanied with a vertically aligned structure, as well as index of refraction that is close to unity,^[^ ^72^ ^]^ which are the distinguishing features of graphene oxide (GO) nanostructures,^[^ ^73^ ^]^ allowed implementing the material as a highly efficient adsorber of solar energy in Arctic regions, or in a robust setup designed to desalinate seawater and remove pollutants from sewage. Several examples of nanowire‐based systems are shown in **Figure** **5**. Vertical graphene can be obtained by a number of plasma methods,^[^ ^74^ ^]^ and functional groups that can be generated on the surface of GO after its functionalization, allows obtaining a high hydrophilicity, which are coupled with intrinsic high efficiency of GO with respect to the photothermal conversion. When operating, the top layer of the partially reduced GO adsorbs solar energy with the efficiency of 92.97%, which is used to evaporate water from the heated tips of the nanostructure, and the hydrophilic surface of the whole structure ensures the delivery of water flow from the bottom part of the device.^[^ ^75^ ^]^ Opposite to that, in desert where water is usually presented in a form of fog, biologically inspired structures also obtainable by use of plasma techniques can be employed.^[^ ^76^ ^]^ Thus, a fibre of a spider web encrusted with glue droplets allows capturing the water droplets from the mist, while cactuses feature a twisted grooved structure with trichomes and the structure gradients intended for water transportation. After analysing the nature‐inspired mechanisms, a yarn composed of carbon nanotubes and ionic silk fibroin glue to mimic the processes that occur in a cactus structure and spider web was developed.^[^ ^77^ ^]^ The structure of the composite allows adsorbing the mist, its liquefaction, and delivery of the collected water to the end of the yarn by use of the effect of capillary lubrication. Silk fibroin and Ca^2+^ ions were combined to produce the glue that is capable to adsorb water from the environment. Moreover, even in water‐scarce regions with absence of fogs, fresh water can be extracted from atmosphere. Nano‐fibrillated cellulose (NFC) integrated with LiCl solution and graphene to form a portable solar‐powered nanostructured biopolymer hygroscopic aerogel (NBHA) for atmospheric water harvesting (AWH) was successfully applied.^[^ ^78^ ^]^ In the material, LiCl particles are immersed into the nano‐fibrillated cellulose to collect water from atmosphere, and this composite ensures the moisture adsorption, while the water release relies on evaporation, which is ensured by the sunlight collected by the upper part of the material (graphene) exposed to the solar irradiation. In addition, DBD plasma coat developed on condensation surface affects greatly on the surface wettability and hydrophobicity, as well as the condensing temperature and freshwater production rate—the latter can be increased by about 30% at the cold plasma coating.^[^ ^79^ ^]^ Soil can also be used in arid regions as a water source under the condition of presence of some minerals. It was discovered that the crystal planes of gypsum rocks can be beneficial for the formation of a biofilm produced by the bacteria, where the minerals are dissolved with the water extraction,^[^ ^80^ ^]^ yet the mechanism of the extraction should be developed.

Microwave plasma is suitable for co‐production of oxygen and NO_x_ for fertilizer, even when employed in Martian atmosphere.^[^ ^81^ ^]^ When extracting water from atmosphere under extremely low air humidity, a tubular solar still black cotton cloth bed filled with calcium chloride desiccant can be employed.^[^ ^82^ ^]^ Moreover, an interesting setup named as “plasma broom” illustrate the possibilities of plasma application in dusty regions where the energy production depends on solar energy.^[^ ^83^ ^]^ A few shots of plasma jet directed to an array of photovoltaic cells covered with dust allows cleaning the cell surfaces with necessary effectiveness, and 5 Torr of Martian environment is beneficial for keeping the plasma flux focused.

Water splitting and Fischer–Tropsch process are not the only ways to produce energy in remote area. Plasma is considered as an alternative to ammonia synthesis,^[^ ^86^ ^]^ and itself is a powerful engineering tool to create the necessary facilities such as microstructured nanogenerators that are capable for vibration energy harvesting in a wide bandwidth range.^[^ ^87^ ^]^ In addition, plasma technologies are widely applied in the development of alkali‐ion and metal‐based batteries, as well as and supercapacitors.^[^ ^88^ ^]^

Providing residents with food is another problematic issue in isolated habitats. Nowadays, atmospheric cold plasma is considered as a platform technology with a huge potential for many production stages to increase germination and yield,^[^ ^89^, ^90^ ^]^ produce nitrate fertilisers, reduce bacterial levels at harvest, for pesticides degradation,^[^ ^91^ ^]^ pest and mycotoxin removal, sterilization of food.^[^ ^92^ ^]^ Moreover, DBD plasma can be engaged for palm oil hydrogenation allows producing margarine with formation of low trans‐fatty acids.^[^ ^93^ ^]^ Plasma is also an emerging method for water purification and recycling,^[^ ^94^ ^]^ as well as a tool for the management of water quality in aquaculture and aquaponics.^[^ ^95^, ^96^ ^]^

## 4 Architecture

**Figure 6:**
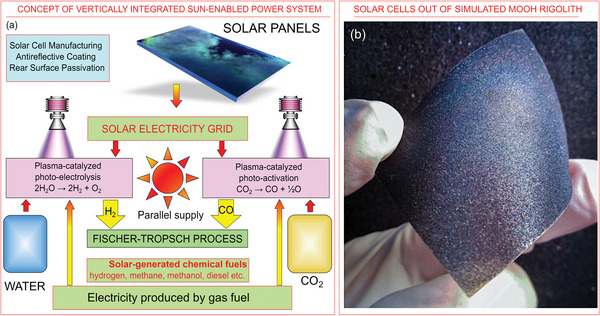
Concept of vertically integrated Sun‐enabled power system. a) The proposed self‐contained system, dreamed for remote habitats including Moon and Mars settlements. The initial energy is supplied form the Sun and then distributed to the immediate customers and the plasma‐activated processes to produce CO, hydrogen and oxygen which is vitally required the Moon and Mars settlements for breathing and other purposes, apart from the energy production. The Fischer–Tropsch process is the used to produce liquid fuels that are very efficient energy storage media. Importantly, the system includes the production of solar cells for direct supply of solar electricity to the customers and accumulation system in the main energy generation unit. b) The first experiments on the production of solar cells out of the material similar to lunar regolith is shown in (b): an optical photograph of the semi‐finished monograin layer solar cell to be produced on the Moon. Reproduced with permission from Kristman et al. 2022.^[^ ^97^ ^]^ The efficient plasma‐catalysed water and carbon dioxide splitting systems, producing syngas and then liquid fuel via the FTP are the core of the energy conversion sub‐system. Next, liquid fuel is the mass‐efficient energy storage system.

The ideally self‐contained system, dreamed, e.g., remote habitats and Mars settlements, could be assembled of the following subunits and processes (**Figure** **6**):

(i) Production (regeneration) of c‐Si solar cells for direct supply of solar electricity to the customers/accumulation system, utilizing Sun‐generated and Sun‐produced plasma environment;
(ii) Solar energy harvesting for splitting of H_2_O and CO_2_ to produce H_2_ and CO, respectively (not only using the solar cell produced electricity, but when possible, using Sun radiation for direct production of plasma, as illustrated in Figure 2a, and the following utilization plasma for efficient plasma‐enabled reactions);
(iii) Production of other types of chemicals and fuels using energy from H_2_ and CO via the established Fischer–Tropsch (FT) process, followed by the generation of electric energy from these fuels and its supply to consumers when the energy directly generated by solar cells is not available, or insufficient (e.g., conducting some energy‐demanding operations).

Highly efficient c‐Si solar cells and parallel supply of H_2_ and CO (via FT process) by solar power are the key processes, while the flexible control of non‐equilibrium plasma is the key feature of the approach.

Environmentally friendly and low‐cost plasma processes in manufacturing of c‐Si solar cells have been intensively developed for the last decade. Two‐step plasma enhanced process based on application of inductively coupled plasma (ICP) setup was developed for production of nanotextured photovoltaic cells, where the first stage is conducted by use of nitrogen plasma to perform doping of c‐Si substrates for junction formation, and the second engaged argon‐hydrogen plasma mixture to make nanotexturing and defect passivation.^[^ ^98^ ^]^ Capacitively coupled SiH_4_ plasma (CCP) was utilized for fabrication nanocrystalline Si films, when the ultra‐crystallinity of the film is controlled by the reactor operation parameters.^[^ ^99^ ^]^ Atmospheric plasma spray system was implemented to deposit TiO_2_ hollow micro‐ and nanospheres used as feedstock for preparation of a surface with photocatalytic properties.^[^ ^100^ ^]^ Plasma enhanced chemical vapour deposition (PECVD) allowed the growth multiwalled carbon nanotubes by utilizing liquefied petroleum gas as carbon precursor,^[^ ^101^ ^]^ as well as via other approaches.^[^ ^102^ ^]^ Oxygen plasma pre‐treatment was necessary to remove organic admixtures, when fabricating the devices suitable for photovoltaics, light‐emitting, and field‐effect applications on a base of terpene green solvents obtained from renewable feedstocks.^[^ ^103^ ^]^

Plasma‐production of antireflection coatings with graded index for reduction of light reflectance can significantly improve the effectiveness of the integral management of photon absorption and electron–hole pair collection in c‐Si solar cells. Remarkable decrease in reflectance of a hydrogenated silicon nitride layer intended for c‐Si solar cells was achieved after its treatment in argon plasma; moreover, the layer was grown in PECVD process conducted in a mixture of NH_3_ and H_2_ gases.^[^ ^104^ ^]^ When fabricating c‐Si nano‐structured solar cells, one‐step process based on implementation of ICP discharge was necessary to synthesize simultaneously and anti‐reflection and n‐layer by etching the p‐type silicon wafers in argon‐hydrogen plasma with optimized parameters.^[^ ^105^ ^]^ A roll‐to‐roll plasma sputtering system was employed to produce antireflective and self‐cleaning plasma‐polymerized fluorocarbon layer to be incorporated to perovskite solar cells.^[^ ^106^ ^]^ Arrays of ZnO 1D nanostructures were fabricated in RF PECVD setup with a purpose to serve as an antireflective surface in c‐Si solar cells where the efficiency of 20% in photovoltaic conversion was reached.^[^ ^107^ ^]^ Multi‐layered architecture developed by use of PECVD was studied to decrease the value of optical losses, and the lowest reflectivity of 1.05% was achieved for six layers based on silicon nitride and silicon oxynitride.^[^ ^108^ ^]^

The recycling of carbon dioxide (CO_2_) into synthetic fuels via Power‐to‐Gas (PtG)^[^ ^109^ ^]^ by implementation of plasma reactors in the production cycle is also beneficial. Plasma gasification of potentially contaminated medical waste products combined with water electrolysis, methanol synthesis, supercritical CO_2_ power cycle, and heating subsystems was put in a base of a system intended for methanol–electricity cogeneration with an energy efficiency more than 60%.^[^ ^110^ ^]^ DBD plasma reactor provided with a solid sorbent was developed for capture and splitting carbon dioxide; plasma ignition allowed not only controlling the process of conversion into carbon monoxide with the efficiency of more than 40%, but also served as an interrupter of the adsorption process, which suggests the process as a promising tool for syngas production.^[^ ^111^ ^]^

## 5 Outlook: Further Steps and Challenges

The discovery of water ice on Mars is considered as a major breakthrough during the decades of the intensive researches carried on the Red Planet. A mission of Mars Express Orbiter^[^ ^112^ ^]^ performed by the European Space Agency was successful in finding the liquid water under the Martian south pole. In addition, traces of buried ice were distinguished among the data delivered from the Shallow Radar (SHARAD) installed on Mars Reconnaissance Orbiter.^[^ ^113^ ^]^ All these discoveries raise the hope that water can be found in the diverse sources on Mars, instead of being transported from Earth, which will greatly contribute to the settlement on Mars.

Thus, efficient energy harvesting, transformation and storage indeed will be in demand in the nearest future. Apart from the described above general architecture and outlined availability of key technologies for building such system, what are possible directions for the further enhancing the efficiency?

**Figure 7:**
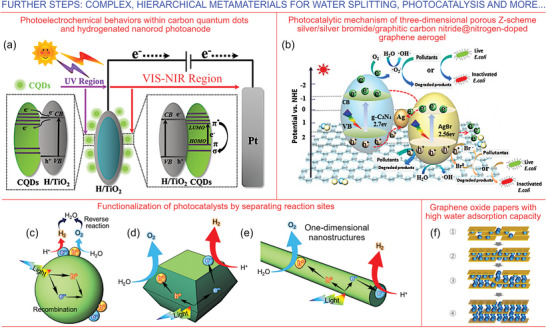
Further steps—complex, hierarchical metamaterials for water splitting, photosynthesis and other processes and reactions related to the advanced energy systems. a) A complex metamaterial comprising hydrogenated TiO_2_ nanorods decorated with carbon quantum dots for the efficient photoelectrochemical water splitting. Illustration of enhanced photochemical process in carbon quantum dots (CQDs)‐H/TiO_2_ photoanodes. The hydrogenation reaction results in the generation of oxygen vacancies and Ti^3+^ cations in TiO_2_ photoanodes to hinder the recombination of charge carriers; the quantum dots greatly intensify the process of harvesting of solar radiation. The developed photoanodes show the efficiency of ≈66.8% for the incident photon to current conversion, thus achieving almost sixfold increase with respect to the performance of pristine TiO_2_. Reprinted with permission.^[^ ^54^ ^]^ Copyright 2019, American Chemical Society. b) Photocatalytic mechanism of three‐dimensional porous Z‐scheme silver/silver bromide/graphitic carbon nitride@nitrogen‐doped graphene aerogel. This complex material demonstrates excellent visible‐light photocatalytic and antibacterial activities. Reprinted with permission.^[^ ^117^ ^]^ Copyright 2019, Elsevier. To enhance the efficiency of water splitting photocatalysts, the recombination of exitons should bve suppressed by separation of holes and electrons via (c) random loading of co‐catalysts; (d) formation of facets; (e) with one‐dimensional nanostructures. Reprinted under the terms of the CC BY license.^[^ ^118^ ^]^ Copyright 2022, The Authors. f) Schematic of water molecules adsorption inside the GO flakes in the graphene oxide‐based paper featuring high water adsorption capacity. Graphene oxide paper used for packaging of fruits increased post‐harvest lifespan of produce by improving moisture adsorption and mitigating mold growth, suggesting future uses of this material in moisture management and in food safety context. Reprinted under the terms of the CC NY license.^[^ ^119^ ^]^ Copyright 2022, The Authors.

The answer could be—complex, hierarchical, and nature‐inspired nanomaterials and metamaterials.^[^ ^114^, ^115^ ^]^ For example, complex architectures with oriented graphene can be used for water harvesting and purification—as a step to derive clean water for splitting (**Figure** **7**).^[^ ^116^ ^]^

While the described system could be quite efficient and very suitable for remote locations where logistics will definitely represent a significant problem (e.g., delivery of equipment, reagents, and consumables to Mars and Moon settlements or very remote areas on the Earth), the stability, long service life and the possibility of regeneration of various sub‐systems and materials should be considered as a very important factor and carefully examined during the development of the general architecture and specific design solutions for the system. Importantly, local resources such as chemicals present in soils, regolith, atmosphere, and underground (e.g., water is expected to be available on Mars^[^ ^120^ ^]^ and even on the Moon^[^ ^121^ ^]^) should be carefully evaluated for the possible application in the system, and where possibly, the system design and the involved processes should be adapted to the available local resources.

Selection, stability and regeneration of catalysts could represent a particularly serious problem since the quality of catalyst directly influences the efficiency and cost of the output.^[^ ^122^ ^]^ Here we should note that a wide variety of catalytic systems could be used as catalysts for the TF systems, thus making it possible for the system to rely on the available local resources.^[^ ^123^ ^]^

The very wide range of catalysts including iron‐based systems (Fe‐alkali/SiO_2_, K/silica, Fe‐Cu/MnO_2_, and other), Cobalt‐based (Co/Ceria, Co/Titania, Co/Alumina, and other), Ruthenium catalysts^[^ ^124^ ^]^ and others that could be used in such a system allows for greater controllability and flexibility in respect to the local resources.

**Figure 8:**
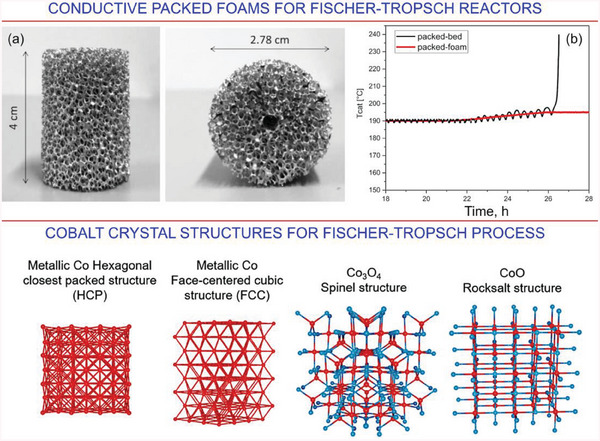
TOP PANEL: Further steps—novel materials for catalytic systems. a) Optical photographs of the open‐cell aluminium foams. b) Dependence of catalyst temperature on time for the packed foam and packed bed reactors. Reprinted with permission under the terms of the CC BY‐NC‐ND license.^[^ ^126^ ^]^ BOTTOM PANEL: Examples of cobalt crystal structures used for the FT process. Reprinted with permission^[^ ^128^ ^]^

**Figure 9:**
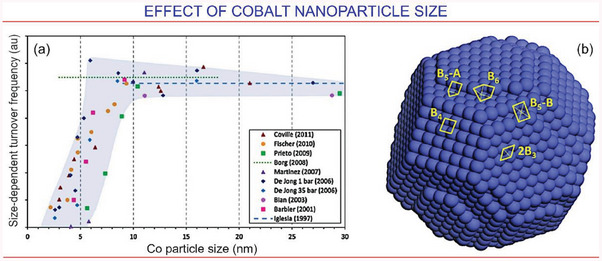
Effect of cobalt nanoparticle size in FT process. a) The relationship between the size of cobalt nanoparticle and turnover frequency. Data from van de Loosdrecht et al.,^[^ ^130^ ^]^ van Helden et al.,^[^ ^131^ ^]^ and elsewhere.^[^ ^132^, ^133^, ^134^, ^135^, ^136^, ^137^, ^138^ ^]^ b) Multiple distinct Bn sites present on a single cobalt nanoparticle (face‐centred‐cubic structure, particle size: 4 nm). Note two types of reactive B5 sites. Reproduced with permission.^[^ ^131^ ^]^ Reproduced with permission.^[^ ^128^ ^]^

Indeed, very common chemical elements such as Fe could be used as catalysts for the TF systems.^[^ ^125^ ^]^ Furthermore, recent achievements in the design of high‐performance, nanotechnology‐based catalytic systems based on these elements open up opportunities to also ensure very high efficiency and stability of the TF systems,^[^ ^125^ ^]^ thus addressing the logistics problem. As some examples, we can show the recently demonstrated conductive aluminum foams packed with catalyst microspheres (**Figure** **8**a,b).^[^ ^126^ ^]^ Another example is the catalytic composition for the FT process made of µm‐sized carbon spheres with cobalt clusters.^[^ ^127^ ^]^ Moreover, properly selected crystalline structures of the active catalytic material (Figure 8) and the size of catalytic nanoparticles (**Figure** **9**) can be used to significantly influence the outcome.^[^ ^128^, ^129^ ^]^

Further development of advanced nanomaterial‐based catalysts for the FT processes is ongoing and will continue to increase the efficiency of catalysts to further reduce the cost associated with their delivery and replacement at remote places, especially if the premise of using local resources for the regeneration is built into their design at the concept stage.

Optimization of the general design of the system also could contribute to its stability by ensuring more efficient energy generation in, e.g., three‐stage systems.^[^ ^139^, ^140^ ^]^

We should stress here one feature of the FT process that usually is considered as a disadvantage of this scheme, but at remote locations that need to produce various materials instead of delivering them (say to Mars and Moon outposts) may become a significant advantage. Specifically, the polymerization type mechanism that drives the FT synthesis typically leads to a very wide range of products in the yield. This relative lack of selectivity toward a particular product is considered as a significant limitation of this technology because additional unit operations are required to maximise the yield of a target product.

**Figure 10:**
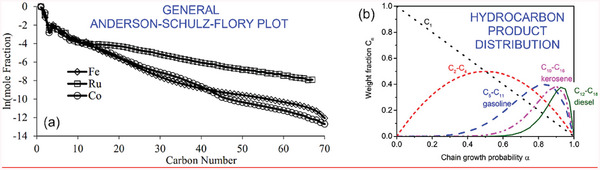
a) Linear plot of the distribution of products generated using the FT synthesis, where vertical axis is the ln of the mole fraction and horizonal axis is carbon number. An assumption is made of carbon chain lengths being dependent only on the molar rates of chain propagation and termination. Fe, Ru, and Co are used as catalysts. Reproduced under the terms of the CC BY license.^[^ ^124^ ^]^ b) Hydrocarbon product distribution. Reproduced with permission.^[^ ^128^ ^]^

Indeed, Anderson et al.,^[^ ^141^ ^]^ Schulz,^[^ ^142^ ^]^ and Flory^[^ ^143^ ^]^ have independently arrived at the model (now called the Anderson–Schulz–Flory (ASF) model) to define the possible hydrocarbon products that can be produced during the FT synthesis. Equation (1) defines the ideal distribution of products of the FT synthesis as:

(1) $$\frac{M_{n}}{n}={\left(1-\alpha \right)}^{2}\;\alpha^{n-1}$$

where *M*_n_ */n* defines the mole fraction of a hydrocarbon with carbon number *n*, and α is the probability of chain growth which in turn is dependent on the relationship between the molar rate of chain propagates and that of chain termination. **Figure** **10** shows the product distributions for iron, cobalt, and ruthenium catalysts when *n* is an independent variable.^[^ ^124^ ^]^

The composition of the output can be controlled over a wide range through optimisation of the process design. Recent studies demonstrate a significant progress toward increasing the controllability of this process, and no carbon formation has been detected experimentally nor with modelling in the advanced Fisher–Tropsch reactor.^[^ ^144^ ^]^

While this may be considered a disadvantage for conventional chemical plants, this flexibility may offer additional possibilities for the remote habitats where a wide range of products is necessary for various aims, supplying the settlement occupants with many high‐demand materials via a single processing platform.

## Conflict of Interest

The authors declare no conflict of interest.
